# The non-fatal burden of cancer in Belgium, 2004–2019: a nationwide registry-based study

**DOI:** 10.1186/s12885-021-09109-4

**Published:** 2022-01-13

**Authors:** Vanessa Gorasso, Geert Silversmit, Marc Arbyn, Astrid Cornez, Robby De Pauw, Delphine De Smedt, Ian Grant, Grant M. A. Wyper, Brecht Devleesschauwer, Niko Speybroeck

**Affiliations:** 1 Department of Epidemiology and Public Health, Sciensano, Rue J Wytsman 14, 1050 Brussels, Belgium; 2 Department of Public Health and Primary Care, Ghent University, Ghent, Belgium; 3 Research Department, Belgian Cancer Registry, Brussels, Belgium; 4 Department of Human Structure and Repair, Faculty of Medicine and Health Sciences, Ghent University, Ghent, Belgium; 5 Department of Rehabilitation Sciences, Ghent University, Ghent, Belgium; 6 Public Health Scotland, Edinburgh, Scotland; 7 Department of Translational Physiology, Infectiology and Public Health, Ghent University, Merelbeke, Belgium; 8 Institute of Health and Society (IRSS), Catholic University of Louvain, Brussels, Belgium

**Keywords:** Burden of disease, Cancer, Years lived with disability

## Abstract

**Background:**

The importance of assessing and monitoring the health status of a population has grown in the last decades. Consistent and high quality data on the morbidity and mortality impact of a disease represent the key element for this assessment. Being increasingly used in global and national burden of diseases (BoD) studies, the Disability-Adjusted Life Year (DALY) is an indicator that combines healthy life years lost due to living with disease (Years Lived with Disability; YLD) and due to dying prematurely (Years of Life Lost; YLL). As a step towards a comprehensive national burden of disease study, this study aims to estimate the non-fatal burden of cancer in Belgium using national data.

**Methods:**

We estimated the Belgian cancer burden from 2004 to 2019 in terms of YLD, using national population-based cancer registry data and international disease models. We developed a microsimulation model to translate incidence- into prevalence-based estimates, and used expert elicitation to integrate the long-term impact of increased disability due to surgical treatment.

**Results:**

The age-standardized non-fatal burden of cancer increased from 2004 to 2019 by 6 and 3% respectively for incidence- and prevalence-based YLDs. In 2019, in Belgium, breast cancer had the highest morbidity impact among women, followed by colorectal and non-melanoma skin cancer. Among men, prostate cancer had the highest morbidity impact, followed by colorectal and non-melanoma skin cancer. Between 2004 and 2019, non-melanoma skin cancer significantly increased for both sexes in terms of age-standardized incidence-based YLD per 100,000, from 49 to 111 for men and from 15 to 44 for women. Important decreases were seen for colorectal cancer for both sexes in terms of age-standardized incidence-based YLD per 100,000, from 105 to 84 for men and from 66 to 58 for women.

**Conclusions:**

Breast and prostate cancers represent the greatest proportion of cancer morbidity, while for both sexes the morbidity burden of skin cancer has shown an important increase from 2004 onwards. Integrating the current study in the Belgian national burden of disease study will allow monitoring of the burden of cancer over time, highlighting new trends and assessing the impact of public health policies.

**Supplementary Information:**

The online version contains supplementary material available at 10.1186/s12885-021-09109-4.

## Background

One of the key challenges health care decision makers are confronted with is how to allocate available resources to optimally address the population health needs [1]. An evidence-based answer to this question involves an evaluation of the health status of the population, ideally based on coherent and comparable measures of morbidity and mortality. Confronted with this need, there has been an increased interest in the establishment of burden of disease studies [2–4]. At international level, the World Health Organization (WHO) and the Institute for Health Metrics and Evaluation (IHME) have set the standard for Global Burden of Disease (GBD) studies [5, 6]. At the national level, several countries, including Belgium, have initiated national or regional burden of disease studies, aiming to make best use of country-specific available data and knowledge [2, 3, 7, 8]. Central to the framework of the global and national burden of disease studies, the Disability-Adjusted Life Year (DALY) metric quantifies the healthy life years lost due to living with disease (Years Lived with Disability; YLD) and due to dying prematurely (Years of Life Lost; YLL) [9].

In Belgium, as in many other high-income countries, cancer is a major contributor to the overall burden of disease [10]. Thanks to early diagnosis and more effective therapies, the long-term survival of some cancer patients increased over the years [11], e.g., breast cancer 5-year net survival in high income countries is now 85–90% [12]. Nevertheless, the disease still affects the independence in performing daily living activities, also due to treatment-related disabling complications (e.g., breast cancer-related lymphedema, axillary web syndrome) [11] and psychosocial distress [13]. To date, however, country-specific estimates of the morbidity burden of cancer in terms of YLD are lacking, despite the existence of an exhaustive cancer registry. The latter represents a source for estimates that are more specific and sensitive to the Belgian framework, compared to currently available international estimates. In addition, quantifying the non-fatal burden is important for assessing the burden that is not derived by death, e.g. of people living longer with their disease. The aim of this study was, therefore, to estimate the non-fatal burden of cancer in Belgium based on locally available data and knowledge.

## Methods

Our study concerns a nationwide registry-based analysis, estimating the non-fatal burden of cancer in Belgium using both an incidence and a prevalence perspective. The incidence perspective takes new diagnoses as a starting point, and quantifies the future health losses due to disability. Incidence-based burden of disease estimates can be used to monitor the impact of preventive measures. The prevalence perspective quantifies the health losses for the current cancer cases, and can therefore be used to assess the burden to the healthcare system. We adopted the 10-year prevalence in line with the GBD study [5], i.e. counting all individuals that had a cancer diagnosis in the past 10 years as prevalent cases.

In what follows, we describe the two main elements of this study – i.e., the data source for cancer incidence and survival, and the disease models used to translate incidence and survival into prevalence, and cases into YLD.

Belgian cancer registry data

### Incidence data

Data on new cancer cases in Belgium are collected by the Belgian Cancer Registry (BCR). The BCR is a population-based registry regularly reporting on cancer patterns and trends in incidence and cancer survival. It is nationally representative and exhaustive, collecting data from the oncological care programs (clinical network) and pathology laboratories (pathological network) [14]. The recording of data (topography and morphology) is done using the International Classification of Diseases for Oncology third edition (ICD-O-3), which is combined into a ICD-10 classification (International Classification of Diseases tenth edition). The vital status was derived from linkage with the Belgian Crossroads Bank for Social Security. Follow-up for this study was considered up to April 1st, 2020.

For the current study, we selected 80 ICD-10 (C00.0–96.9 and chronic myeloid neoplasms) codes resulting in 54 cancer groups (see Appendix). Data were extracted by year (from 2004 to 2019), age group (5-years), sex and region (*N* = 3). We excluded “Respiratory system and intrathoracic organs, NOS (not otherwise specified)” from further analyses because of too few cases.

#### Survival data

To assess the time lived with disability, we calculated observed survival estimates by large age groups (< 50, 50–64, and 65+), region, and 10-year incidence period (i.e., from 2004 to 2013 to 2010–2019), according to the Kaplan-Meier method [15]. For the most common cancer types (breast, bronchus and lung, colorectal, malignant melanoma of skin and prostate), smaller age groups were used: < 50, 50–64, 65–69, 70–74, 75–79, 80–84, and 85+. If the age group specific estimates were not available due to too few cases, we used the all-ages estimate instead. Likewise, if the region-specific estimate was not available, we used the Belgian estimate. In some instances, survival estimates were not available for year 9 or 10 after diagnosis, since no patients in that selection had a follow-up of minimum 9/10 years, so the estimate at the time point(s) does not exist. In these cases, we propagated the last observed (annual) survival probabilities to the years without estimates using the interval specific survival (e.g., if no estimates were available for year 10, the survival probabilities were estimated as the product of the survival probability at year 9 and the ratio between the survival of year 9 and the survival of year 8). Per definition, the observed survival estimates for the last available 10-year period (2010–2019) were applied to all cases diagnosed in 2009 or later.

### Disease models

We adopted the disease models used in the most recent GBD study [5]. The models make a distinction between surviving cases, and cases that die within 10 years after diagnosis. For surviving cases, the disease models define two health states 1) diagnosis and primary therapy; and 2) control phase when the cancer becomes a chronic diseases and requires daily medication that do not interfere with daily activity. The duration of the diagnosis stage is cancer specific and the duration of the control stage is given by the remainder of the 10-year period [5]. For fatal cases, the disease models define four health states – i.e., diagnosis, control, metastasis, and terminal. The duration of each stage depends on both the cancer type and the survival time. The durations are assigned in the following sequence:

1. Terminal: 1 month
2. Diagnosis: cancer specific duration (or remainder of total survival time)
3. Metastasis: 18 months (or remainder of total survival time)
4. Control: remainder of total survival time

The disability weights (DW) assigned to these four health states are derived from Salomon et al. (2015); diagnosis and primary treatment (0.288), control (0.049), metastasis (0.451), and terminal (0.540) [16].

#### Inclusion of complications

For some cancers, the disease models also included specific treatment or surgery-induced complications for the entire duration of illness. These complications comprised mastectomy (breast cancer; DW = 0.036), stoma (colorectal cancer; DW = 0.095), laryngectomy (larynx cancer; DW = 0.051), incontinence (prostate and bladder cancer; DW = 0.139), and impotence (prostate cancer; DW = 0.017).

To assess the proportion of cases for which these complications occur, we performed an expert elicitation exercise among experts in contact with our institution. Belgian oncologists, gynecologists and urologists from different hospitals and clinics in Belgium were contacted through email. Each expert was asked to provide a minimal and maximal plausible value for the proportion of complications among the specific cancers for which they had most expertise. The elicitation was done through an online questionnaire. We summarized the expert values into an overall estimate of the proportion of complications per cancer, by computing the average value across experts (see Additional file 1).

### Statistical analyses

#### Estimation of prevalence from incidence

Based on the disease models, we projected the time spent in the different health states for each incident cohort (2004–2019). This implies that from the year 2013 onwards, we were able to define the prevalence in a given year as the sum of person-months spent in the different health states. We used the observed survival probabilities to model the fraction of surviving vs non-surviving cases, as well as the moment of death (in terms of time since diagnosis) for the non-surviving cases. Specifically, we used a microsimulation approach to simulate future health states for each year-, age-, sex-, region- and cancer-specific cohort of incident cases. For each incident case in the specific cohort, age at diagnosis was randomly assigned using a uniform random number generator taking the minimum and maximum of the concerned age group as limits. Then, we used sampling with replacement to assign, for each incident case in the specific cohort, one of 11 possible outcomes according to the survival probabilities, i.e., death within year 1, 2, …, 10 after diagnosis, or survival. For the fatal cases, simulated to die within year y after diagnosis, we randomly assigned the moment of death using a uniform random number generator taking y − 1 and y as limits. The age at death was thus a function of the randomly assigned age at diagnosis, and the randomly assigned time between diagnosis and death. In a final step, we assigned the health states of the cancer disease model to each incident case, in function of their simulated outcome, and, for the fatal cases, their simulated time till death. The durations of each health states, and the sequence in which the health states are defined, were explained before.

#### Incidence-based YLD

Incidence-based YLD were estimated for the period 2004–2019. For each reference year, the YLD were calculated as the sum of the future disability-weighted time spent in each health state, for the cases that were diagnosed in the reference year. The calculation of the amount of time spent in each health state followed the same steps as in the incidence-to-prevalence model, except that we used average values instead of a microsimulation approach.

#### Prevalence-based YLD

Prevalence-based YLD were estimated for the period 2013–2019. For each reference year, the YLD were calculated as the sum of the disability-weighted time spent in each health state, for all the cases that were alive during the reference year.

### Presentation and availability of estimates

Results were presented by age, sex, and region using cancer groupings. We report in this paper the crude rates and age-standardized rates, using Belgian 2019 population as reference.

For more detailed results, the complete cancer burden estimates, including age-standardized rates based on the Belgian population and the European standard population of 2013 [17], can be explored online via https://burden.sciensano.be/shiny/cancer/.

## Results

Incidence-based cancer burden, 2004–2019

### All cancers

From 2004 to 2019, the total number of tumor diagnoses has increased from 61,524 to 80,524 new diagnoses (+ 31%). This is mainly due to the growth and ageing of the population; because, over the same period, the age-standardized incidence rates slightly increased from 649 to 702 new diagnoses per 100,000 (+ 8%). Similar trends are observed for the total number of cancer-associated YLDs, which have increased from 44,774 YLDs to 57,317 YLD (+ 25%), over a period of 15 years, and a slight increase in the age-standardized YLD rates, from 472 per 100,000 to 501 per 100,000 (+ 6%).

**Fig. 1:**
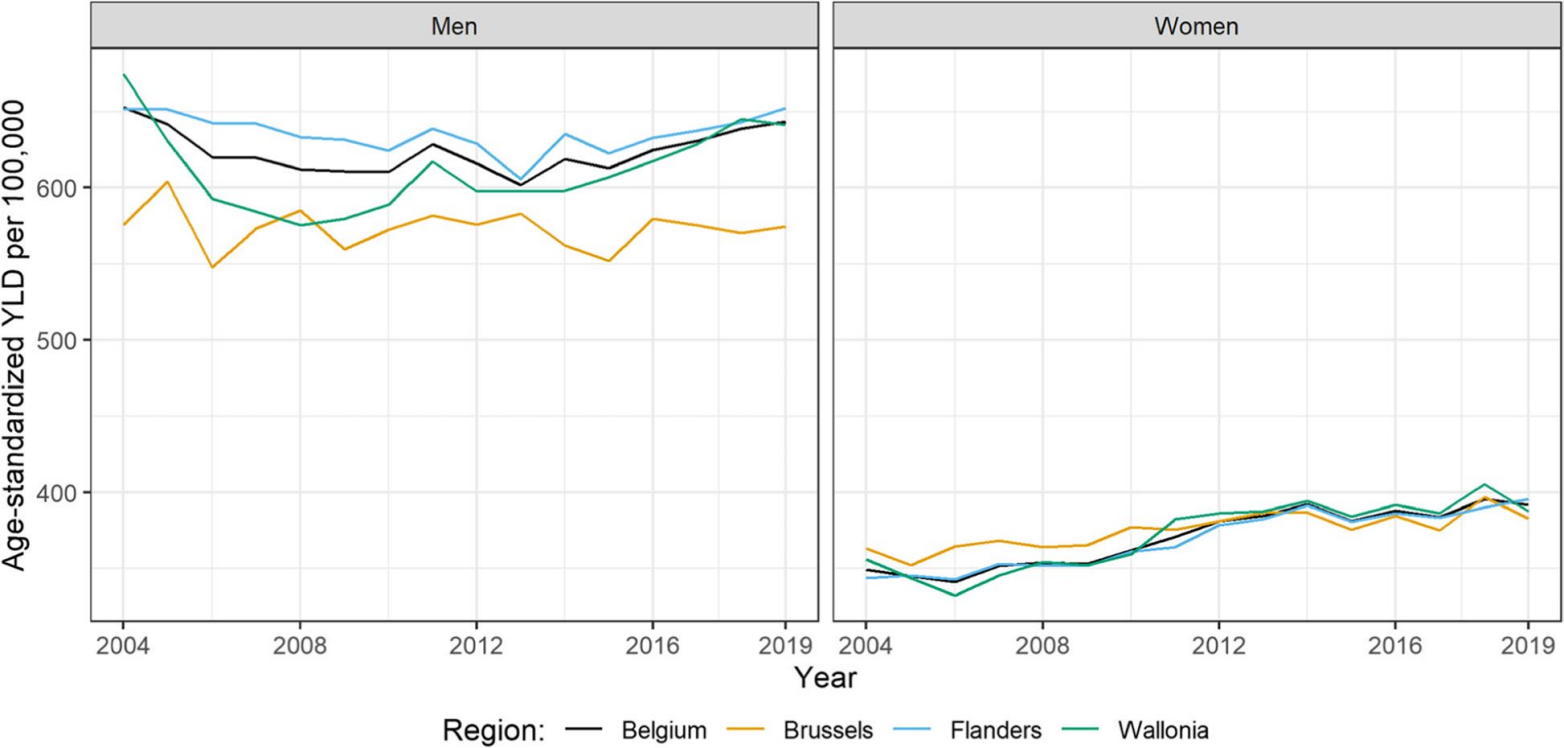
Age-standardized incidence-based YLD per 100,000 rate for all cancers in Belgium and its regions by sex

Cancer incidence and burden were higher in men compared to women, and the highest in the 65+ age group. The age-standardized cancer incidence and burden were the highest in the Walloon Region, followed by the Flemish and Brussels Capital Region. However, due to the larger and older population, the largest absolute cancer burden was attributed to the Flemish Region (Fig. 1).

### Specific cancer types

#### Top 5 cancer types

**Fig. 2:**
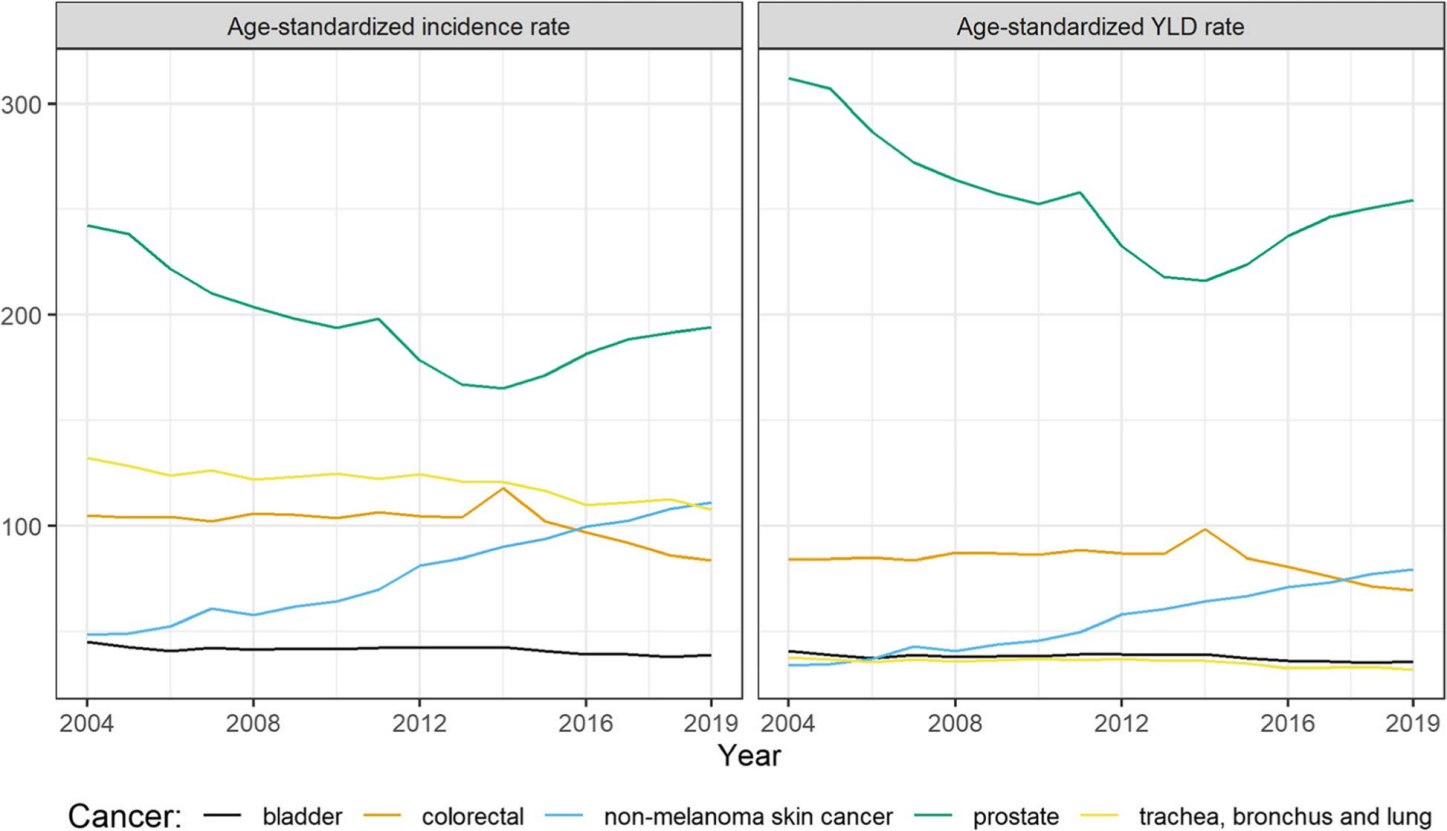
Age-standardized incidence rates and incidence-based YLD for top 5 cancers diagnosed in men from 2004 to 2019

In 2019, the highest number of cancer diagnoses among men were observed for prostate cancer, trachea, bronchus and lung cancer, non-melanoma skin neoplasms, colorectal cancer, and bladder cancer. The same cancers can be found in the top-5 ranking in terms of YLD burden. Prostate cancer remained at the first place followed by non-melanoma skin neoplasms, colorectal cancer, bladder cancer and trachea, bronchus and lung cancer. As shown in Fig. 2, almost all top 5 cancers showed a decrease in their YLD burden, with prostate cancer showing the largest decrease from 2004 (312 vs 254 age-standardized YLD per 100,000) followed by colorectal cancer (105 vs 84 age-standardized YLD per 100,000). Non-melanoma skin neoplasms showed a steady increase since 2004 (49 vs 111 age-standardized YLD per 100,000).

**Fig. 3:**
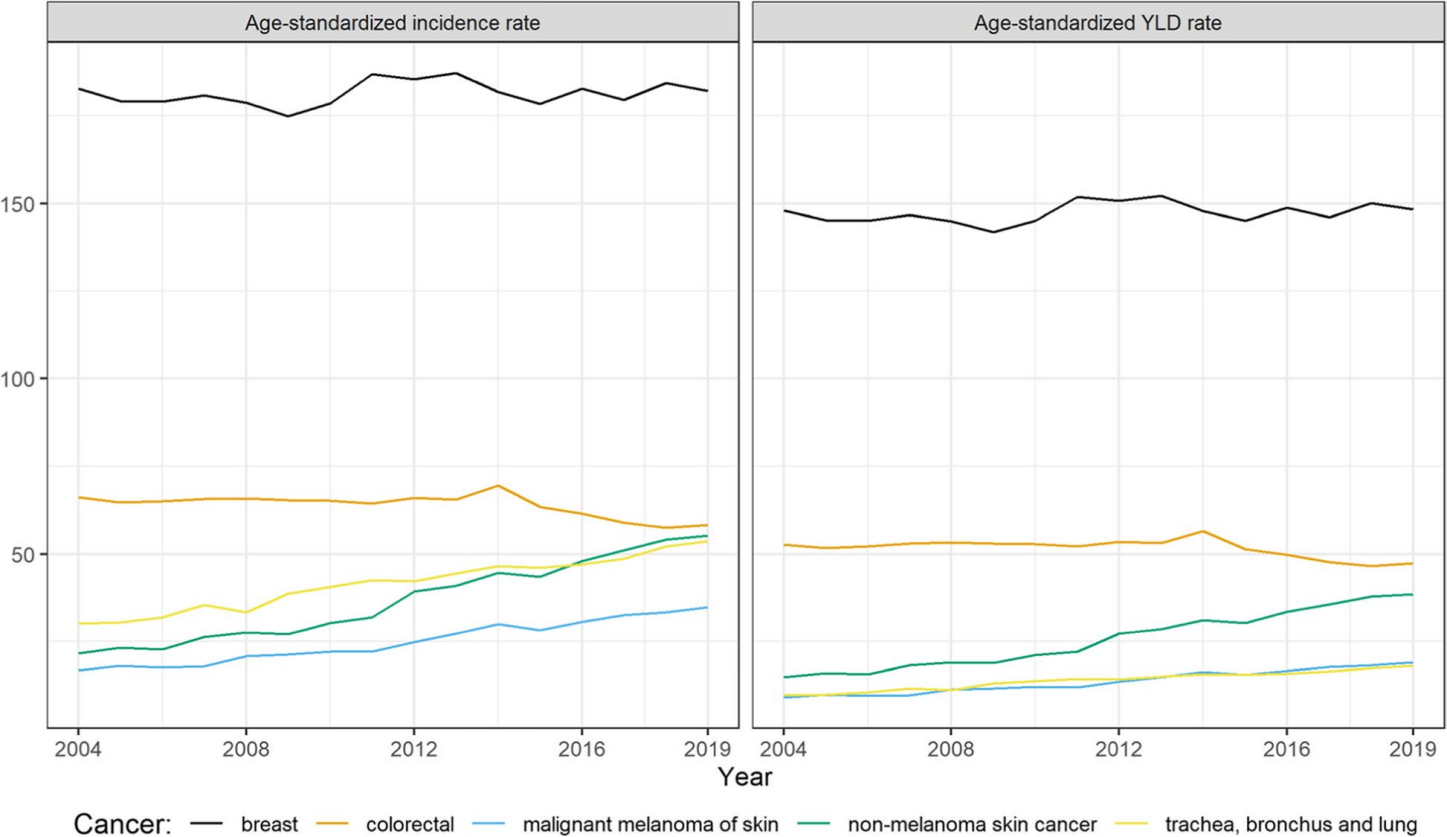
Age-standardized incidence rates and incidence-based YLD for top 5 cancers diagnosed in women from 2004 to 2019

In 2019, the highest number of cancer diagnoses among women were for breast cancer, colorectal cancer, non-melanoma skin neoplasms, trachea, bronchus and lung cancer, and malignant melanoma of skin, corresponding also to cancers with the highest non-fatal burden. Figure 3 shows that different cancers types showed an important increase since 2004. The non-fatal burden of malignant melanoma of skin doubled passing from 9 to 19 age-standardized YLD per 100,000 and non-melanoma skin neoplasms more than doubled (15 vs 44 age-standardized YLD per 100,000). Trachea, bronchus and lung cancer also showed large increase (30 vs 54 age-standardized YLD per 100,000). Colorectal cancer was the only top-5 cancer to show a decrease in the observed period (66 vs 58 age-standardized YLD per 100,000).

When looking at both sexes, rankings across regions looked rather similar. However, prostate cancer has a higher burden in Flanders than in the two other regions, together with skin cancers. Breast, colorectal and lung cancer showed a higher non-fatal burden in Wallonia. For both sexes it is also noticeable the reduction in colorectal cancer cases of the last 4 years.

#### Other cancers

Along with the most burdensome cancers, it is worth mentioning some cancers that have particularly striking trends from 2004 to 2019. Liver and pancreas cancer respectively almost tripled and doubled in terms of incidence in the observed period. On the other hand, many gynecological cancers showed a reduction: uterus NOS (− 87%), ovarian (− 24%) and vaginal (− 13%). Nevertheless, the reduction in new diagnosis of uterus NOS cancer might be driven by a better quality of data reporting. Namely, cancer diagnosis are more correctly attributed to cervix and corpus uteri, leading to a reduction of cancers coded as uterus NOS.

### Prevalence-based cancer burden, 2013–2019

#### All cancers

From 2013 to 2019, the yearly prevalence has increased from 379,742 to 432,106 (14%). An increase of the age-standardized prevalence rates from 3581 to 3770 per 100,000 can also be seen over the same period (5%). A similar trend was observed for the total number of cancer associated prevalence-based YLD, with an increase from 45,887 to 51,464 YLD (12%), reflected in an increase in the age-standardized YLD rates from 435 per 100,000 to 449 per 100,000 (3%).

**Fig. 4:**
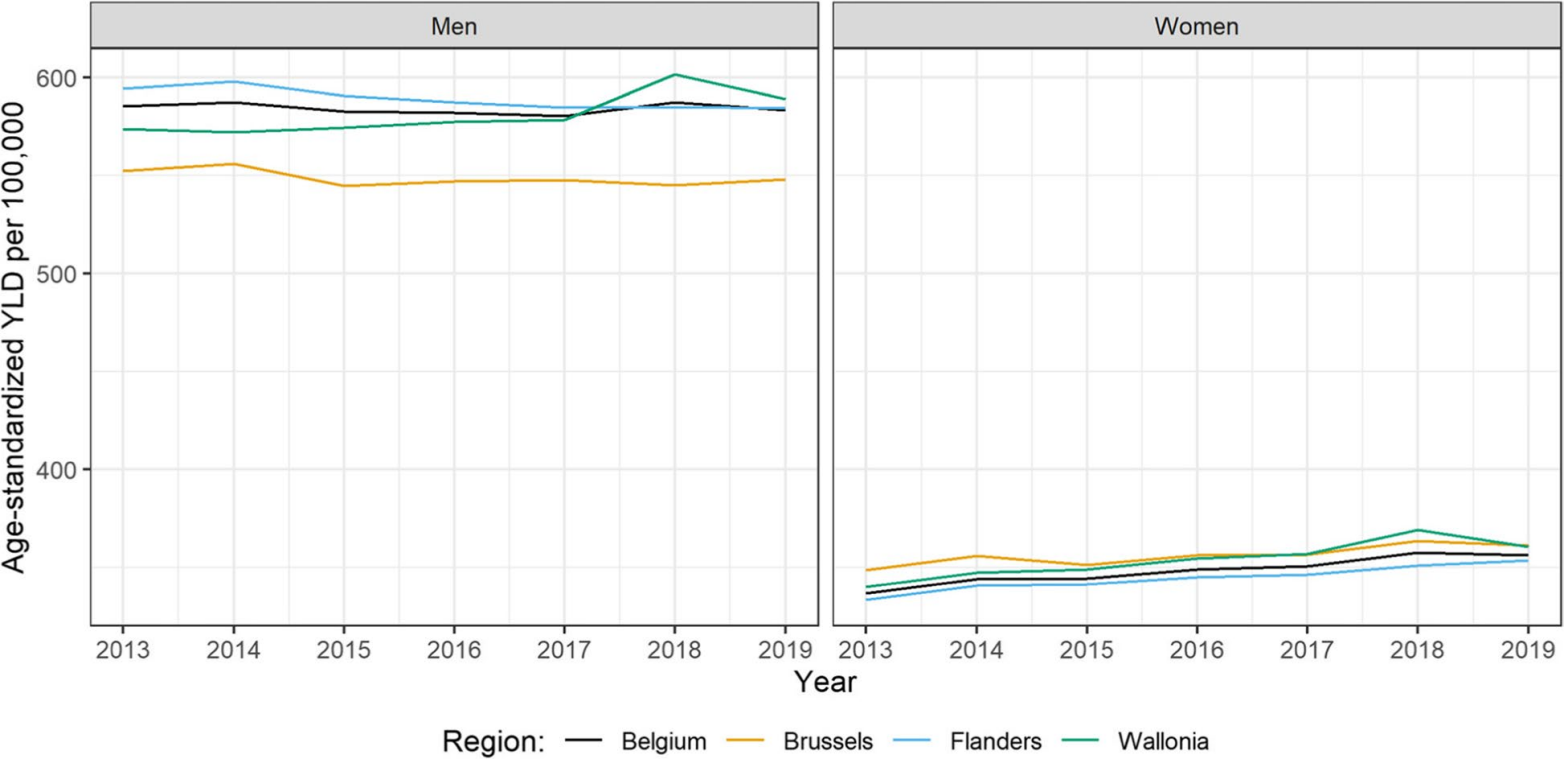
Age-standardized YLD prevalence-based YLDs per 100,000 rate for all cancers in Belgium and its regions by sex

In 2019, the all-cancers age-standardized prevalence rate was higher in men than in women (4228 and 3527 per 100,000 persons respectively) and both sexes showed a considerable prevalence in the 65+ age group. Due to the larger and older population, the Flemish Region was responsible for the largest absolute cancer burden. However, when we look at the age-standardized rates, the Walloon Region had the highest prevalence and YLD per 100,000 rates (Fig. 4). We can also notice that Wallonia and Brussels showed an increase in prevalence (for both sexes) in the last five years.

### Specific cancer types

#### Top 5 cancer types

**Fig. 5:**
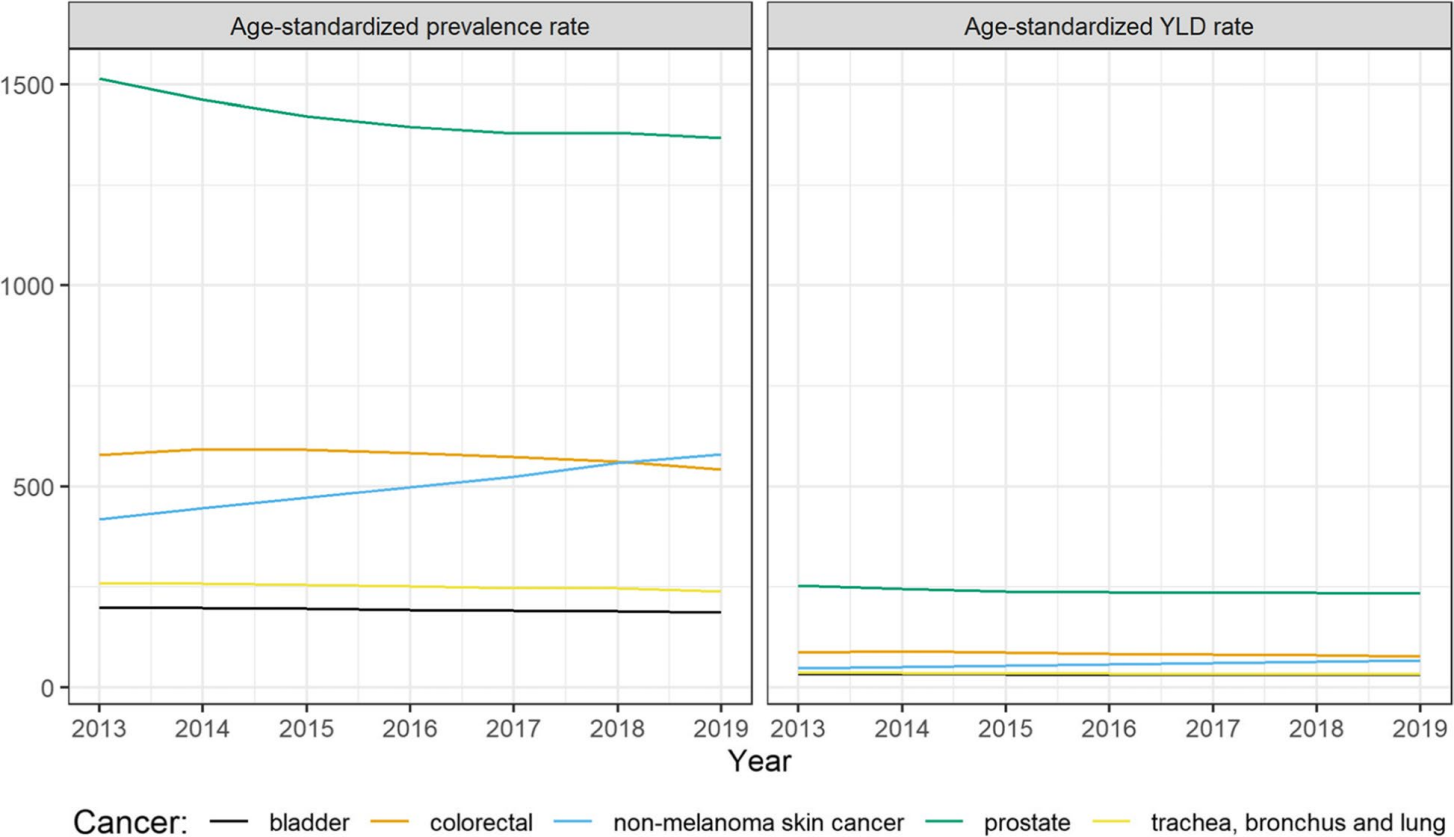
Age-standardized prevalence and prevalence-based YLD for top 5 cancers diagnosed in men from 2013 to 2019

In 2019, the most common cancers registered among men were prostate cancer (1248 per 100,000 persons), colorectal cancer (490 per 100,000 persons), non-melanoma skin cancer (472 per 100,000 persons), trachea, bronchus and lung cancer (224 per 100,000 persons), and malignant melanoma of skin (179 per 100,000 persons). The same cancers were identified as having the highest non-fatal burden, except for bladder cancer that passed to be within the top 5 (replacing malignant melanoma of skin). Figure 5 shows the trends of the top 5 cancers in men between 2013 and 2019. A decrease was observed in the age-standardized prevalence rate for prostate cancer (from 1515 to 1368). On the other hand, non-melanoma skin cancer showed an increase over the years with passing from 417 to 581 per 100,000 persons. The same trends are reflected in the non-fatal burden of these cancers.

**Fig. 6:**
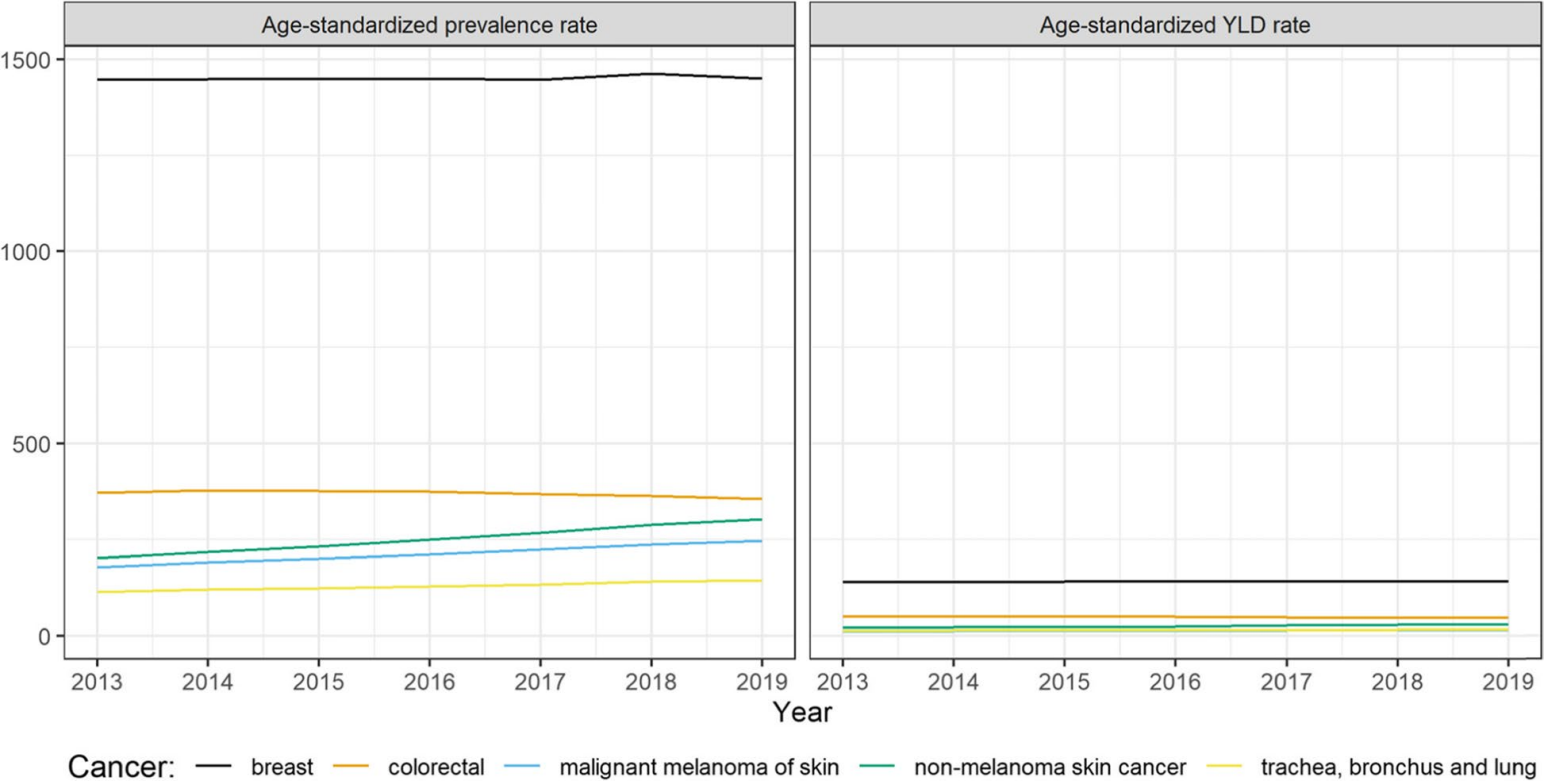
Age-standardized prevalence and prevalence-based YLD for top 5 cancers diagnosed in women from 2013 to 2019

In 2019, breast cancer was the most prevalent cancer in women (1501 per 100,000 persons), followed by colorectal cancer (387 per 100,000 persons), non-melanoma skin cancer (348 per 100,000 persons), malignant melanoma of skin (252 per 100,000 persons) and corpus uteri (174 per 100,000 persons). The same order was reflected for the ranking of the cancers with the highest non-fatal burden, apart from the fifth cancer that was replaced by lung cancer. As shown in Fig. 6, between 2013 and 2019 there has been a decrease in the age-standardized prevalence rate for colorectal cancer (from 372 to 355 per 100,000 persons) and corpus uteri cancer (from 175 to 161 per 100,000 persons). On the other hand, skin cancers showed an increase over the years with malignant melanoma passing from 179 to 247 per 100,000 persons and non-melanoma skin cancer from 202 to 303 per 100,000 persons. The same trends were reflected in the non-fatal burden of these cancers.

When looking at both sexes, the most prevalent cancer in Belgium in 2019 was breast cancer: 815 per 100,000 age-standardized persons in Brussels, 795 in Wallonia and 750 in Flanders. However, the cancer type with the highest non-fatal burden was prostate cancer in 2019: 109 per 100,000 age-standardized YLD in Flanders, 95 in Wallonia and 86 in Brussels.

#### Other cancers

When looking at the non-top 5 cancers, interesting changes in the 5-year time span can be observed. In 2019, thyroid gland (5 age-standardized YLD rate) and lip and oral cavity cancer (6 age-standardized YLD rate) were both among the cancers with the highest prevalence-based YLDs overall in Belgium, and from 2013 they showed an increase in prevalence of around 22 and 9%, respectively. In terms of prevalence, cancer of the uterus NOS showed the highest relative decrease from 2013: − 74%, probably attributed to the more specific registration of cervix and corpus uteri cancer, as explained above.

## Discussion

In the present study, we estimated the non-fatal burden of 54 cancer groups in Belgium based on data from the national population-based cancer registry. From 2004 to 2019, Belgium experienced an increase in the cancer age-standardized incidence rate as well as in the age-standardized prevalence rate. In 2019, more than 80,000 new cancers were diagnosed and more than 430,000 people were living with cancer. The most incident and prevalent cancer was breast cancer among women, and prostate cancer among men. It is worth mentioning that most of the increase in the age-standardized incidence and prevalence can be attributed to the increase in non-melanoma skin cancer cases. Our results showed the important burden of these cancers in terms of disability with around 50,000 YLD each year. Incidence and prevalence-based YLD estimates do not differ much from each other, but overall prevalence-based YLD rates tended to be lower than incidence-based YLD rates, which is consistent with an increasing cancer incidence and improved survival.

Comparing incidence figures with other national studies, we find that incidence rates for female breast cancer in Belgium were slightly higher than the ones for The Netherlands (184/100,000 vs 153/100,000 women in 2014). In the Netherlands, they registered a steady increase of incident breast cases [18], similarly to our study. GBD 2019 incidence results for Belgium top-5 cancers were, in general, lower than the ones reported in this study (for breast [9700 vs 11,057], for prostate [8100 vs 10,196] and lung cancer [8676 vs 8886]), with the exception of colorectal cancer [8993 vs 7990] [5]. Nevertheless, our estimates represent the closest estimation to the real values, considering the population-based cancer registry data (see below for accuracy of dataset) and that no modelling was applied.

The main patterns of cancer morbidity burden in Belgium do not differ much from other European countries. According to a joint burden of cancer study, prostate, colorectal, breast and lung cancer are the most frequently diagnosed cancers in Europe [19]. Spanish national estimates also identified colorectal, breast and lung cancer as accounting for the most YLD. In particular colorectal cancer accounted for 16% of all YLD due to cancer in Spain in 2000 [3]. Breast, colorectal and prostate resulted also to have the highest number of age-standardized YLD rate in Italy, with prostate and breast having among the highest YLD contribution to DALY [4]. In addition, our study confirms the increase of skin cancer for both men and women in Belgium, as shown in a previous national study, which also estimated that the skin cancer burden and associated economic impact in Belgium would triple in the next 20 years [20]. When comparing our morbidity estimates with the GBD 2019 results, we noticed that the GBD estimates are generally lower compared to our figs [5]. In particular, lung and non-melanoma skin cancer were much lower with a prevalence of 21,360 and 46,882 in our estimates, versus 12,260 and 1700 in the GBD study, respectively. GBD prostate estimates were also lower than in the study at hand, with YLD estimates of 5460 versus 11,770 in our study. This comparison highlights the importance of producing national estimates.

The large non-fatal disease burden of cancer suggests that there are still considerable opportunities for improving the health burden related to malignancies in Belgium. In addition, our estimates highlight the differences among regions that might shift the focus of the interventions (e.g. Belgian regions have competencies regarding health prevention). Epidemiological trends show that cancer was, is and probably will continue to be a major contributor to the national burden of disease. National policies should further focus on reducing cancer incidence and preventing disability. For example, lifestyle interventions, including diet and physical activity when combined with chemotherapy can enhance treatment efficacy [21, 22], or different types of counseling, psycho-education or therapy can help with cancer-related fatigue [23].

### Strengths and limitations

Our study compiled epidemiological data and burden of disease estimates for the great majority of cancers by cancer site. The BCR represents a reliable data source for neoplasm estimates in Belgium. The completeness of the BCR is estimated to be more than 95% and the validity of the data is ensured by having very high percentage of tumors being microscopically verified (96.9%) [24, 25].

Despite the good quality of the incidence data, our estimates come with uncertainty associated with the disease models and estimation processes. The health state durations were adopted from the GBD study, and might not be representative for the Belgian context. Moreover, by adopting the disease model in the GBD study, we assumed that all cancers have the same DW. For the sake of internal consistency, we decided to follow GBD methodological choices. Nevertheless, national/region-specific survival durations were used, that already improve certainty over part of the process [26]. The proportions of specific surgery or treatment-induced complications was obtained through expert elicitation, a process which resulted in considerable uncertainty. To address these limitations, methods should be explored to obtain data-driven estimates of complication probabilities, for instance based on the national health insurance data managed by the Intermutualistic Agency (IMA). Moreover, when including the sequelae related to treatment in the disease models, we assumed that they would last for the entire duration of the disease. This might yielded an overestimation of the YLDs, since the complication-inducing surgery or long-term treatments might take place weeks or months (but not years) after initial diagnosis. Finally, we did not perform a formal uncertainty quantification of our estimates, mainly because not all sources of uncertainty could be quantified.

## Conclusion

Cancer has a major impact on the health of the Belgian population. Breast and prostate cancers represent the greatest proportion of cancer morbidity, while for both sexes the morbidity burden of skin cancer has shown an important increase from 2004 onwards. Integrating the current study in the Belgian national burden of disease study will allow monitoring the burden of cancer that can affect the availability of healthcare treatment and service accessibility. Such results can be also used to highlight new trends and assess the impact of public health policies.

### Future perspectives

The project includes yearly updates of the non-fatal burden of cancer, available via https://burden.sciensano.be/shiny/cancer/. In addition, we aim to complement these non-fatal burden estimates with fatal burden estimates (Years of Life Lost), derived from the national mortality database maintained by Statistics Belgium. Furthermore, the research team is in the process of setting up an analysis concerning the direct healthcare cost associated to cancer, including BCR data on diagnosis and cost data provided by IMA.

## Supplementary Information

Additional file 1

## Data Availability

All data generated or analysed during this study are included in this published article and can be found in https://burden.sciensano.be/shiny/cancer/

## Abbreviations

BCR: Belgian cancer registry
DALY: Disability-adjusted life years
DW: disability weight
GBD: Global Burden of Disease
ICD-10: International Classification of Diseases tenth edition
ICD-O-3: International Classification of Diseases for Oncology third edition
IMA: Intermutualistic agency
IHME: Institute for Health Metrics and Evaluation
NOS: not-otherwise specified
WHO: World Health Organization
YLD: Years Lived with Disability
YLL: Years of Life Lost
