# Supplementary material for: The non-fatal burden of cancer in Belgium, 2004–2019: a nationwide registry-based study
Source: BMC Cancer. 2022 Jan 13;22:58. doi: 10.1186/s12885-021-09109-4 (PMC8756629; doi:10.1186/s12885-021-09109-4)
Supplement: Supplementary file 1 — Additional file 1 [file 12885_2021_9109_MOESM1_ESM.docx]

**Supplementary material**

Appendix 1: Categorization of cancer groups

| **ICD-10** | **Name** | **Group name (if any)** |
| --- | --- | --- |
| C00 | Lip | Lip and oral cavity |
| C01 | Base of tongue |  |
| C02 | Tongue |  |
| C03 | Gum |  |
| C04 | Floor of mouth |  |
| C05 | Palate |  |
| C06 | Mouth, NOS |  |
| C07 | Parotid gland |  |
| C08 | Salivary glands, NOS |  |
| C09 | Tonsil | Other pharynx cancer |
| C10 | Oropharynx |  |
| C11 | Nasopharynx |  |
| C12 | Pyriform sinus |  |
| C13 | Hypopharynx |  |
| C14 | Lip, oral cavity and pharynx, NOS |  |
| C15 | Oesophagus |  |
| C16 | Stomach |  |
| C17 | Small intestine |  |
| C18 | Colon |  |
| C19 | Rectosigmoid junction |  |
| C20 | Rectum |  |
| C21 | Anus and anal canal |  |
| C22 | Liver and intrahepatic bile ducts |  |
| C23 | Gallbladder | Gallbladder and biliary tract |
| C24 | Biliary tract, NOS |  |
| C25 | Pancreas |  |
| C26 | Other ill-defined digestive organs |  |
| C30 | Nasal cavity and middle ear |  |
| C31 | Accessory sinuses |  |
| C32 | Larynx |  |
| C33 | Trachea | Tracheal, bronchus and lung cancer |
| C34 | Bronchus and lung |  |
| C37 | Thymus |  |
| C38 | Heart, mediastinum and pleura |  |
| C39 | Respiratory system and intrathoracic organs, NOS |  |
| C40 | Bone and articular cartilage of limbs |  |
| C41 | Bone and articular cartilage, NOS |  |
| C43 | Malignant melanoma of skin |  |
| C44 | Malignant neoplasms of skin |  |
| C45 | Mesothelioma |  |
| C46 | Kaposi's sarcoma |  |
| C47,C49 | Soft tissues |  |
| C48 | Retroperitoneum and peritoneum |  |
| C50 | Breast |  |
| C51 | Vulva |  |
| C52 | Vagina |  |
| C53 | Cervix uteri |  |
| C54 | Corpus uteri |  |
| C55 | Uterus |  |
| C56 | Ovary |  |
| C57 | Female genital organs, NOS |  |
| C58 | Placenta |  |
| C60 | Penis |  |
| C61 | Prostate |  |
| C62 | Testis |  |
| C63 | Male genital organs, NOS |  |
| C64 | Kidney | Kidney |
| C65 | Renal pelvis |  |
| C66 | Ureter |  |
| C67 | Bladder |  |
| C68 | Urinary organs, NOS |  |
| C69 | Eye and adnexa |  |
| C70 | Meninges | Brain and nervous system |
| C71 | Brain |  |
| C72 | Spinal cord, cranial nerves and CNS, NOS |  |
| C73 | Thyroid gland |  |
| C74 | Adrenal gland |  |
| C75 | Endocrine glands, NOS |  |
| C76 | Other and ill-defined sites |  |
| C80 | Unknown primary site |  |
| C81 | Hodgkin lymphoma |  |
| C82-C86 | Non-Hodgkin-lymphoma | Non-Hodgkin-lymphoma |
| C88 | Malignant immunoproliferative diseases | Multiple myeloma |
| C90 | Multiple myeloma |  |
| C91 | Lymphoid leukaemia | Leukemia |
| C92 | Myeloid leukaemia |  |
| C93 | Monocytic leukaemia |  |
| C94-C95 | Leukaemia other |  |
| C96 | Lymphoid, haematopoietic and related tissue, NOS | Non-Hodgkin-lymphoma |
| MDS | Myelodysplastic syndromes |  |
| MPN | Myeloproliferative neoplasms |  |
|  |  |  |

Appendix 2: Duration by cancer type

| **Cancer** | **ICD10** | **Diagnosis** | **Metastasis** |
| --- | --- | --- | --- |
| Lip | C00 | 5.3 | 9.33 |
| Tonsil | C09 | 5.3 | 7.91 |
| Oropharynx | C10 | 5.3 | 7.91 |
| Nasopharynx | C11 | 5.3 | 13.19 |
| Pyriform sinus | C12 | 5.3 | 7.91 |
| Hypopharynx | C13 | 5.3 | 7.91 |
| Oesophagus | C15 | 5.0 | 4.60 |
| Stomach | C16 | 5.2 | 3.88 |
| Colon | C18 | 4.0 | 9.69 |
| Rectosigmoid junction | C19 | 4.0 | 9.69 |
| Rectum | C20 | 4.0 | 9.69 |
| Anus and anal canal | C21 | 4.0 | 9.69 |
| Liver and intrahepatic bile ducts | C22 | 4.0 | 2.51 |
| Gallbladder | C23 | 4.0 | 3.47 |
| Biliary tract, NOS | C24 | 4.0 | 3.47 |
| Pancreas | C25 | 4.1 | 2.54 |
| Larynx | C32 | 5.3 | 8.84 |
| Bronchus and lung | C34 | 3.3 | 4.51 |
| Malignant melanoma of skin | C43 | 2.9 | 7.18 |
| Mesothelioma | C45 | 4.0 | 7.75 |
| Breast | C50 | 3.0 | 17.70 |
| Cervix uteri | C53 | 4.8 | 9.21 |
| Uterus | C54 | 4.6 | 11.60 |
| Ovary | C56 | 3.2 | 25.60 |
| Prostate | C61 | 4.0 | 30.35 |
| Testis | C62 | 3.7 | 19.47 |
| Kidney | C64 | 5.3 | 5.38 |
| Renal pelvis | C65 | 5.3 | 5.38 |
| Bladder | C67 | 5.1 | 5.80 |
| Meninges | C70 | 5.0 | 6.93 |
| Brain | C71 | 5.0 | 6.93 |
| Spinal cord, cranial nerves and CNS, NOS | C72 | 5.0 | 6.93 |
| Thyroid gland | C73 | 3.0 | 19.39 |
| Hodgkin's disease | C81 | 3.7 | 26.00 |
| Non-Hodgkin-lymphoma | C82-C85 | 3.7 | 7.70 |
| Malignant immunoproliferative diseases | C88 | 7.0 | 36.82 |
| Multiple myeloma | C90 | 7.0 | 36.82 |
| Lymphoid leukaemia | C91 | 6.0 | 48.00 |
| Myeloid leukaemia | C92 | 6.0 | 48.00 |
| Monocytic leukaemia | C93 | 6.0 | 48.00 |
| Leukaemia other | C94-C95 | 6.0 | 48.00 |
| Lymphoid, haematopoietic and related tissue, NOS | C96 | 3.7 | 7.70 |
| Other |  | 4.4 | 15.81 |

Appendix 3: Expert elicitation for the proportion of complications

| **Complication** | **Min** | **Max** | **Expert ID** | **Expertise** | **Comments** |
| --- | --- | --- | --- | --- | --- |
| Mastectomy (female) |  | 95% | 6 | Gynecology-obstetrician | Normally, 95% of patients with breast cancer have surgery, man or women. The ones with metastasis from the start are not always operated |
| Mastectomy (female) | 20% | 30% | 9 | Medical oncologist |  |
| Mastectomy (female) | 95% |  | 19 | Gynecology-obstetrician | Only the metastatic patients are not operated. Sure you include partial mastectomy = tumorectomy |
| Mastectomy (female) | 0% | 50% | 22 | Gynecologic oncology | Goal less than 40 % |
| Mastectomy (male) |  | 95% | 6 | Gynecology-obstetrician | Normally, 95% of patients with breast cancer have surgery, man or women. The ones with metastasis from the start are not always operated |
| Mastectomy (male) | 50% | 60% | 9 | Medical oncologist |  |
| Mastectomy (male) | 95% |  | 19 | Gynecology-obstetrician | Only the metastatic patients are not operated. Sure you include partial mastectomy = tumorectomy |
| Mastectomy (male) | 90% | 100% | 22 | Gynecologic oncology | Due to the size of the male breast, a breast sparing is rarely performed |
| Mastectomy (male) | 90% | 100% | 30 | Breast surgeon |  |
| Difficult speech (larynx cancer) | 60% | 70% | 9 | Medical oncologist |  |
| Difficult speech (larynx cancer) | 30% | 40% | 15 | Medical oncologist | Highly variable according to clinical stage and treatment given. |
| Difficult speech (larynx cancer) | 50% | 90% | 24 | Head & neck oncologist | Depends on treatment modality |
| Difficult speech (larynx cancer) | 40% | 80% | 27 | Medical oncologist |  |
| Ileo- or colostomy (colorectal cancer) | 10% | 15% | 9 | Medical oncologist |  |
| Ileo- or colostomy (colorectal cancer) | 5% | 15% | 11 | Digestive oncologist |  |
| Ileo- or colostomy (colorectal cancer) | 10% | 20% | 15 | Medical oncologist |  |
| Ileo- or colostomy (colorectal cancer) | 10% | 20% | 18 | Gastro-enterologist |  |
| Impotence (prostate cancer) | 60% | 70% | 9 | Medical oncologist |  |
| Impotence (prostate cancer) | 30% | 80% | 10 | Urologist |  |
| Impotence (prostate cancer) | 60% | 80% | 15 | Medical oncologist | Excluded those who were impotent BEFORE cancer treatment |
| Impotence (prostate cancer) | 25% | 80% | 26 | Urologist |  |
| Impotence (prostate cancer) | 80% | 100% | 27 | Medical oncologist |  |
| Impotence (prostate cancer) | 65% | 90% | 28 | Uro-oncologist |  |
| Incontinence (prostate cancer) | 50% | 60% | 9 | Medical oncologist |  |
| Incontinence (prostate cancer) | 15% | 70% | 10 | Urologist |  |
| Incontinence (prostate cancer) | 20% | 40% | 15 | Medical oncologist |  |
| Incontinence (prostate cancer) | 5% | 35% | 26 | Urologist |  |
| Incontinence (prostate cancer) | 40% | 50% | 27 | Medical oncologist |  |
| Incontinence (prostate cancer) | 5% | 50% | 28 | Uro-oncologist | Is usually temporary following radical prostatectomy (50%, <1 year), in 5-10% this is permanent |
| Incontinence (bladder cancer) | 60% | 70% | 9 | Medical oncologist |  |
| Incontinence (bladder cancer) | 30% | 100% | 10 | Urologist | Depending on types of derivation non continent or continent |
| Incontinence (bladder cancer) | 10% | 20% | 15 | Medical oncologist |  |
| Incontinence (bladder cancer) | 10% | 66% | 20 | Uro-oncologist |  |
| Incontinence (bladder cancer) | 70% | 90% | 27 | Medical oncologist |  |
| Incontinence (bladder cancer) | 10% | 30% | 28 | Uro-oncologist | Different scenario's possibly: after radical cystectomy, most patients have a urostoma which is by definition incontinent. With an orthotopic neobladder 75% is continent. In case of non-muscle invasive bladder cancer (no cystectomy), most patients do not have incontinence. |
